# Integrating Clinical and Socio-Environmental Approaches in Managing Rheumatoid Arthritis With Social Determinants of Health: A Case Study of an Elderly Patient in Rural Japan

**DOI:** 10.7759/cureus.50915

**Published:** 2023-12-21

**Authors:** Ryuichi Ohta, Chiaki Sano

**Affiliations:** 1 Community Care, Unnan City Hospital, Unnan, JPN; 2 Community Medicine Management, Shimane University Faculty of Medicine, Izumo, JPN

**Keywords:** family medicine, systems approach, chronic care model, patient-centered care healthcare disparities, rural health services, geriatric medicine, rheumatoid arthritis

## Abstract

Rheumatoid arthritis (RA) poses significant management challenges, especially in elderly patients living in rural areas with limited access to health care. This case report illustrates an integrative approach to managing RA, emphasizing the interplay of clinical, social, and environmental factors. A 72-year-old woman in a rural Japanese setting presented with progressive, widespread joint pain, initially self-managed with over-the-counter medications. Her condition, complicated by socioeconomic constraints and limited access to health care, necessitated a comprehensive management strategy. Clinical examination revealed bilateral joint tenderness, swelling, and high titers of rheumatoid factor and anti-citrullinated protein antibodies, confirming RA. Treatment included methotrexate and prednisolone, complemented by lifestyle modifications. Interdisciplinary collaboration among healthcare professionals, including nutritionists and physiotherapists, facilitated her management. The patient's care was guided by the chronic care model and the ecological model, addressing her clinical needs and socio-environmental context. This holistic approach resulted in improved clinical outcomes and enhanced quality of life. This case highlights the importance of a patient-centered, multidisciplinary approach in managing RA in rural settings. Integrating clinical management with an understanding of social determinants and patient empowerment is crucial for effective treatment. The case underscores the need for adaptable healthcare strategies that are sensitive to the unique challenges faced by elderly patients in rural communities.

## Introduction

Rheumatoid arthritis (RA) is a chronic and systemic inflammatory disorder, predominantly affecting joints and potentially involving multiple organ systems [1]. Its prevalence among older populations highlights an escalating public health issue. RA causes not only joint deformity and destruction but also markedly diminishes the quality of life through pain, decreased mobility, and related comorbidities [1].

Effectively managing RA is challenging due to the complex role of social determinants of health (SDH). These factors, including socioeconomic status, education, healthcare access, and environmental conditions, significantly affect health outcomes [2]. In rural areas, where resources and healthcare access are typically restricted, these SDHs create distinct challenges [3]. Research indicates that patients in these areas experience delayed diagnoses, fewer treatment options, and insufficient follow-up care, resulting in poorer health outcomes compared to those in urban areas [3,4]. Therefore, it is crucial to understand and contextualize this reality within rural settings. Given this reality, it is imperative to consider thorough assessments and interventions aimed at improving the quality of life for RA patients in rural areas.

This case report thus introduces one case study that delves into the relationship between SDH and RA, offering a comprehensive exploration of how these factors interplay to exacerbate the disease's impact in rural settings. This case study is not merely an examination of the clinical aspects of RA but a narrative that weaves in the principles of family medicine to address these broader social and environmental determinants. This case report underscores the importance of a comprehensive medical management plan that goes beyond pharmacological interventions to include preventative strategies and community-integrated care. This approach is characterized by its patient-centered nature, where patient agency and active participation in the management plan are paramount. The manuscript emphasizes that empowering patients in their care decisions leads to more personalized and effective management strategies integrating family medicine principles into managing chronic conditions like RA.

## Case presentation

A 72-year-old woman reported six months of persistent, widespread joint pain, beginning in her wrists and progressively affecting multiple joints. Despite self-medication with acetaminophen 500 mg and a one-week trial of prednisone 10 mg daily by her primary care physician on suspicion of RA, her pain re-emerged and escalated during dose tapering. She, therefore, sought further management at our community hospital. Her medical history included hypertension and dyslipidemia, but no notable surgical procedures or allergies.

Clinical perspective

**Figure 1:**
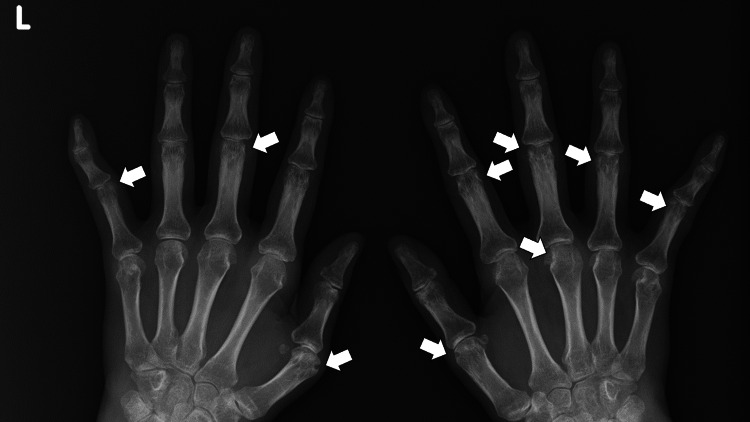
X-ray of bilateral hands showing multiple bone erosions.

The vital signs at the visit were as follows: blood pressure of 156/97 mmHg, pulse rate of 69 beats/minute, body temperature of 37.1°C, respiratory rate of 16 breaths/minute, and oxygen saturation of 98 % on room air. Physical examination revealed bilateral tenderness, swelling, and warmth in the shoulders, wrists, and metacarpophalangeal joints, with absent systemic symptoms. High titers of rheumatoid factor (125 IU/mL; reference < 15) and anti-citrullinated protein antibodies (158 U/mL; reference < 4.5) without any positivity of antinuclear antibodies, coupled with high C-reactive protein (7.2 mg/dl; reference <0.3) and wrists X-ray findings of bone erosion, corroborated the RA diagnosis based on the American College of Rheumatology/European League Against Rheumatism 2010 score of 10 (Figure 1).

Therapeutic prednisolone use prompted the calculation of her risk for secondary hypertension, diabetes, and osteoporosis.

Individual aspect

Despite living independently and engaging in backyard agriculture, the patient’s increasing joint pain, concern about RA progression, and loss of joint function witnessed by her peers became debilitating. Addressing her concerns about disability, the cost of medication, and lifestyle factors worsening her condition, such as a high-carbohydrate diet and sedentary lifestyle, required a comprehensive approach. Her expectations encompassed pain management and the resumption of daily activities without incurring prohibitively expensive treatments.

Contextual dimension

As a widow residing alone in a mountainous region, her non-driving status and limited public transport availability restricted her access to health care and support. While managing on a pension and minimal income from selling vegetables, her children did not support her financially. Furthermore, she found agricultural tasks increasingly difficult due to pain, rendering her income unreliable. These contextual factors significantly influenced her treatment and management strategy.

Treatment and response

The commencement of methotrexate (6 mg/week) and prednisolone (10 mg/day) was complicated by worsening hypertension (150/98 mmHg) and increased blood glucose levels (hemoglobin A1c: 8.6), likely due to prednisolone. The introduction of valsartan (40 mg/day) and metformin (750 mg/day), supplemented by dietary and exercise interventions devised by our multidisciplinary team, stabilized her secondary conditions. A revised higher dose of methotrexate enabled prednisolone discontinuation, further improving glycemic and blood pressure control. The potential complications of methotrexate were followed with monthly laboratory tests, and any abnormality was not observed.

Complications and multidisciplinary approach for rehabilitation and management of chronic diseases

Hyperglycemia and hypertension represented significant challenges to RA management, mandating attentive monitoring and action to mitigate additional risks [1]. Collaboration among internal medicine specialists, nutritionists, and physiotherapists was crucial in managing her complications and ensuring adherence to lifestyle changes. This reflects a scholarly approach to clinical care [2].

Ethical and professional care

Understanding our patient’s fears and financial constraints, our team facilitated access to affordable medication and enlisted community center assistance to ensure she benefitted from social and physical activities. This approach exemplified our dedication to delivering empathetic, ethical, and professionally outstanding care [3].

## Discussion

Applying family medicine principles

Upholding a Patient-Centered Approach

Emphasizing a patient-centered approach in the principle of family medicine, the complexities of managing this elderly woman’s RA became navigable [4]. The five-star family physician framework guided our consideration of her socioeconomic status and family situation, consisting of five competencies such as care provider, decision maker, communicator, community leader, and manager [5]. Her reluctance to engage her children, who reside in distant locales, and her financial constraints shaped our care trajectory, such as focusing on conventional and cost-effective medicines. Recognizing her experiences both medically and personally was crucial for unbiased care provision based on her initial help-seeking behaviors and self-management with over-the-counter medication [5].

Embracing Family-Oriented and Community-Integrated Care

The principle of community-integrated family medicine took precedence, and the spatial and social environment was shared between healthcare providers and the patient [4]. In this case, a shared understanding of prevalent societal issues, such as isolation and challenges in caring for the elderly within a rural milieu, was facilitated. The partnership with the community center post-treatment supported her autonomy and integrated her recovery and ongoing health management with enhanced social connections and a safety net. Consequently, the patient was part of a network allowing her health and social well-being to be cohesively addressed, reflecting family medicine's collaborative and community-integrated ethos.

Comprehensive Management Viewed Through a Preventative Lens

The patient's management transcended the immediacy of RA treatment, venturing into a proactive domain where preventative and therapeutic strategies were interlaced. The sustained use of prednisolone prompted a vigilant approach focusing on early detection and management of complications like hypertension and diabetes [6]. Moreover, including the patient in community health initiatives paid homage to the principle of preventive care and health promotion integrated within the community's fabric and tailored to its peculiarities [4]. The healthcare provided was preventative and health-promotive, tailored to her daily life and circumstances.

Interdisciplinary Collaboration and Management

The merit of interdisciplinary collaboration was illuminated by crafting a symbiotic, patient-centered management path. In line with family medicine principles, family physicians acted as key resource managers, coordinating the involvement of dietitians, therapists, and specialists [4]. Respecting five-star family doctor approaches, this comprehensive, team-driven approach permeated beyond the hospital, assimilating resources from across the medical and community spectrum [5]. The patient's care transcended the domain of medical facilities to intertwine with societal structures. This was foundational in preserving her health and optimizing her quality of life and reflects the role of a manager and leader in holistic patient care [5].

Nurturing Patient Agency and Empowerment

Empowering the patient as an active co-creator in her care journey was paramount [5]. While the family physician’s expertise crafted the medical trajectory, her inputs, derived from her lived experiences, fears, and expectations, informed the management strategy, ensuring it was concurrently clinically sound and empathetically delivered. This approach established a synergy between clinical guidance and the patient’s personal experiences, jointly shaping the care pathway; this promoted adherence and ensured the care was compatible with her daily life rather than an extrinsic, enforced protocol.

Ethical and Professional Adherence

Throughout the course of caring for our patients, professional and ethical adherence was meticulously observed [7]. Her financial constraints and social isolation critically shaped clinical decisions, ensuring they were realistic and practical. The conscientious effort to align medical advice with affordability and accessibility demonstrated an unwavering commitment to equitable, empathetic, and holistic care [8]. Hence, each therapeutic choice and lifestyle recommendation was a testament to ethical, patient-centered medicine.

Social determinants affecting health in the patient and community

Self-Ageism and Its Infiltration Into Healthcare Perceptions

In Japan's elderly, self-ageism often leads to attributing symptoms to aging rather than potentially treatable conditions [9]. This mindset, intensified by Japan's cultural and societal narratives, may result in delays in seeking appropriate medical interventions. It necessitates consideration of symptom management in the context of the patient’s psycho-social dynamics, exploring how societal perspectives of age permeate individual health beliefs and behaviors [10].

Healthcare Accessibility and Literacy

In rural contexts like in some regions of Japan, limitations in healthcare resources and professional accessibility are amplified by gaps in health literacy among the elderly [11]. The barriers extend beyond the physical realm into the digital sphere; health information on the internet is frequently inaccessible to this demographic. Consequently, even with support structures, effective health-seeking behaviors among rural elders may be curtailed, manifesting in delays to or omission of crucial medical interventions for conditions like RA [12].

Social Isolation as a Multi-Faceted Challenge

Rural living, coupled with aging and further complexified by global crises like the COVID-19 pandemic, precipitates physical, emotional, and social isolation [13]. These factors confer an increased risk of poor mental health and physical health implications, such as increased stress and worsening chronic conditions like RA.

Chronic care model

**Figure 2:**
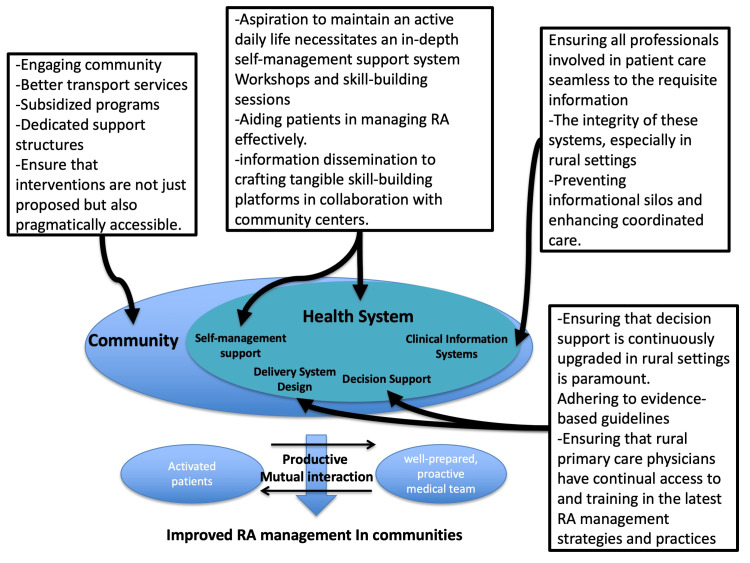
Chronic care model for effective rheumatoid arthritis management in rural contexts RA: rheumatoid arthritis. Image credit: Ryuichi Ohta.

The chronic care model (CCM) is a framework that enhances chronic disease management with an informed, proactive healthcare team and an engaged patient. The model comprises six essential elements: community health system self-management support, delivery system design, decision support, and clinical information systems [14]. This model can be applied to this case and health promotion in her community (Figure 2).

Self-Management Support

The patient’s aspiration to maintain an active daily life necessitates an in-depth self-management support system [14]. The CCM encourages practical steps, such as workshops and skill-building sessions, that could be integral in aiding patients in managing RA effectively [14]. This goes beyond information dissemination to crafting tangible skill-building platforms in collaboration with community centers.

Decision Support

Ensuring that decision support should be implemented and continuously upgraded in rural settings is paramount [14]. This entails adhering to evidence-based guidelines and ensuring that rural primary care physicians have continual access to training in the latest RA management strategies and practices.

Clinical Information Systems and Healthcare Organization

Ensuring that all professionals involved in patient care have seamless access to relevant information underpins effective management [14]. The integrity of these systems, especially in rural settings where healthcare professionals may operate in relative isolation, is crucial for preventing informational silos and enhancing coordinated care.

Community Resources and Policies

While engaging community resources is important, ensuring that these are not just available but also accessible and affordable, especially for elderly individuals in rural settings, is vital. Pragmatically, this may involve transport services, subsidized programs, and dedicated support structures.

Ecological model

**Figure 3:**
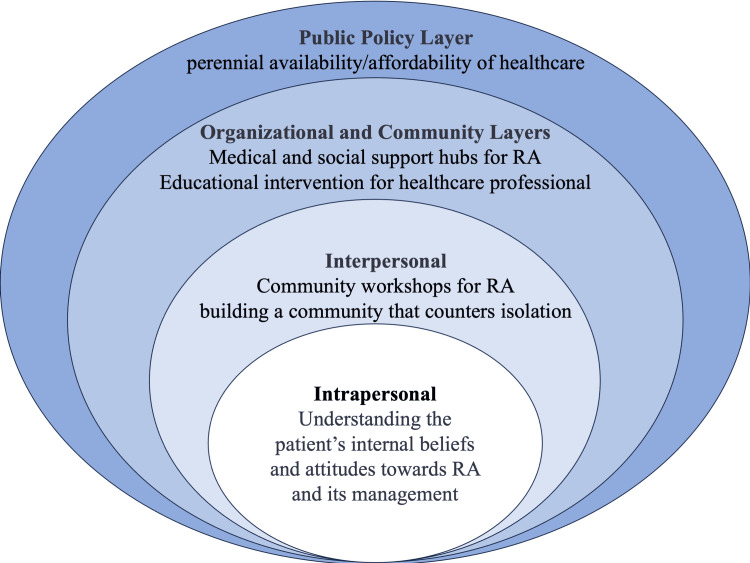
Ecological model for rheumatoid arthritis management in communities RA: rheumatoid arthritis. Image credit: Ryuichi Ohta.

The ecological model emphasizes that interactions between individuals and their environment influence health and well-being. It is broken down into multiple layers: intrapersonal, interpersonal, organizational, community, and public policy [15]. This model can be applied to this case and the health promotion of her community (Figure 3).

Intrapersonal and Interpersonal Layers

Understanding the patient’s internal beliefs about and attitudes toward RA and its management is as crucial as ensuring a supportive social network [15]. Initiatives at this level encompass personal information provision through various information sources such as local newspapers and community workshops [16]. The latter increases understanding of RA management while building a community to counter isolation. Family physicians can collaborate to create articles for local newspapers and establish educational workshops.

Organizational and Community Layers

Local community centers can serve as medical and social support hubs, becoming a nexus for information, resources, and community-building [15]. This is especially pertinent in the social context of rural-dwelling elderly individuals. Family physicians can establish educational workshops for healthcare professionals in rural communities by collaborating with local governments and healthcare institutions [17].

Public Policy Layer

Ensuring that policies are formulated, implemented, and sustained to guarantee healthcare's persistent availability and affordability forms the linchpin on which the other interventions pivot [15]. Family physicians can continually discuss the management of RA in rural communities with policymakers [18].

Comparative insights: CCM vs. ecological model

While the CCM offers a structured approach to chronic disease management, leveraging healthcare team and patient involvement, it may risk overlooking broader social determinants. In contrast, the ecological model provides a multilayered view of the patient within their social and environmental context, suggesting intervention at various societal levels [14,15].

In the context of rural Japan, considerations of aging, social isolation, and healthcare accessibility become paramount [19]. The incorporation of both models offers a holistic approach. While CCM provides a systematic framework for managing RA at a healthcare level, the ecological model ensures that interventions also permeate to the community and policy levels, addressing root social determinants of health [14,15].

Integrating these approaches could curate a care methodology that is not only clinically robust but also socially and contextually sensitive, simultaneously addressing the immediate and root determinants of health [20]. This combined approach bridges immediate healthcare needs with long-term social change, fostering an environment that supports the holistic well-being of the elderly in rural areas.

## Conclusions

Our case study of managing rheumatoid arthritis in an elderly, rural-dwelling woman underscores the need for an integrative approach combining the CCM and the ecological model. This strategy, which addresses both immediate clinical needs and broader socio-environmental factors, proved effective in navigating her complex healthcare and social challenges. The integration of clinical excellence with an understanding of her personal, economic, and community context led to improved health outcomes and quality of life. This case illustrates the importance of a holistic, adaptive approach in rural health care, emphasizing the need for strategies that are both medically robust and socially attuned.
